# Sublingual Dermoid Cyst: A Rare Diagnosis

**DOI:** 10.7759/cureus.67793

**Published:** 2024-08-26

**Authors:** Yash Jain, Apurva Palatkar, Manu Babu, Vinod Shinde, Aditi Moruskar

**Affiliations:** 1 Otolaryngology - Head and Neck Surgery, Dr. D. Y. Patil Medical College, Hospital and Research Centre, Dr. D. Y. Patil Vidyapeeth (Deemed to be University), Pune, IND

**Keywords:** rare case, head and neck cyst, oral cyst, sublingual, dermoid cyst

## Abstract

The head and neck regions are rather uncommon for dermoid cysts. Dermoid cysts are among the rarest types of oral cysts, accounting for a very small proportion of cases in the oral cavity. They are thought to be caused by defective development along the embryonic lines of fusion, involving both ectodermal and endodermal elements. This case study details a 16-year-old girl who complained of a smooth, globular swelling that had been present for two months over the floor of the mouth and extending to her left submandibular region. Following ultrasonography (USG) confirmation, the patient underwent a full surgical excision, and the histological report revealed a sublingual dermoid cyst.

## Introduction

It is rather uncommon to see dermoid cysts in the head and neck regions. Less than 0.01% of oral cysts in the oral cavity are dermoid cysts [1]. They are thought to be the result of defective development along embryonic lines of fusion involving both ectodermal and endodermal elements.

The floor of the mouth is the second most common site where dermoid cyst presentations occur in the head and neck region, behind the periorbital region. Magnetic resonance imaging (MRI), computed tomography (CT) scans, and ultrasonography (USG) are among the diagnostic methods under investigation [2]. The best course of action is to surgically remove the lesion through the mouth [3,4].

Here, we present a unique case of an intraoral dermoid cyst that presented with a pinkish, smooth, globular swelling under the tongue, extending to the submandibular region, and was meticulously managed by surgical excision.

## Case presentation

A 16-year-old girl presented with swelling in the floor of the mouth and left the submandibular area untreated for two months. It was initially small, like the size of a peanut, and gradually progressed to attain the current size of approximately 4×5 cm. She only complained of bulkiness below the tongue. It was not associated with any pain, discharge, or other signs of inflammation. There was no history of any similar complaints, no dysphagia, and no history of trauma, fever, radiation exposure, or other systemic illness. Past and family history were not contributory.

**Figure 1:**
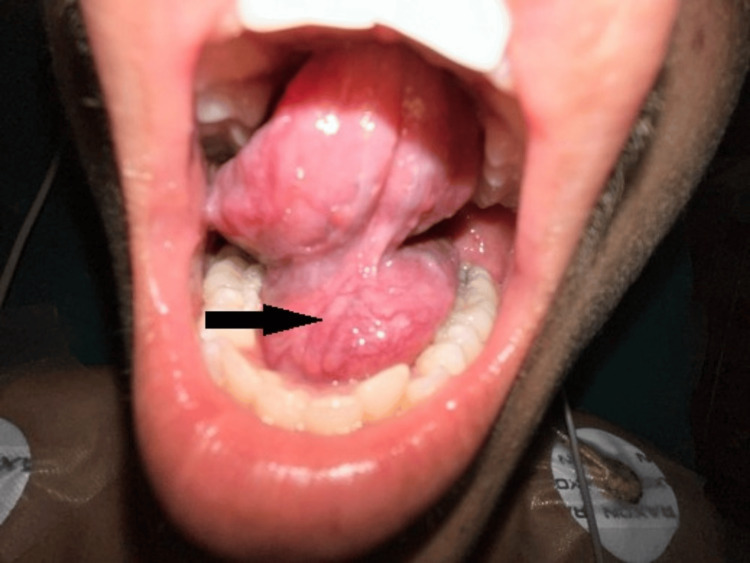
Preoperative clinical picture of a lesion on the floor of the mouth The arrow shows a preoperative picture of the lesion

A 4×3 cm soft-to-firm, pinkish, globular cystic swelling was observed during the clinical examination. It originated from the floor of the mouth on the left side (Figure 1), involved the left submandibular region, and extended to the level of the thyroid cartilage on the left side of the neck. The tongue motions were within normal limits; there was no discharge; the swelling was smooth, non-tender, and soft-to-firm in consistency; and the transillumination test came back negative. The results of the systemic, head and neck, and otorhinolaryngological exams were all normal.

On the USG of the neck, a well-defined, well-circumscribed unilocular cystic lesion, measuring 55×24×52 mm in its largest submandibular location in the left paramedian location, was seen. It showed heterogeneous echotexture, containing numerous small echogenic areas and giving the typical "sack of marbles" appearance suggestive of a dermoid cyst.

**Figure 2:**
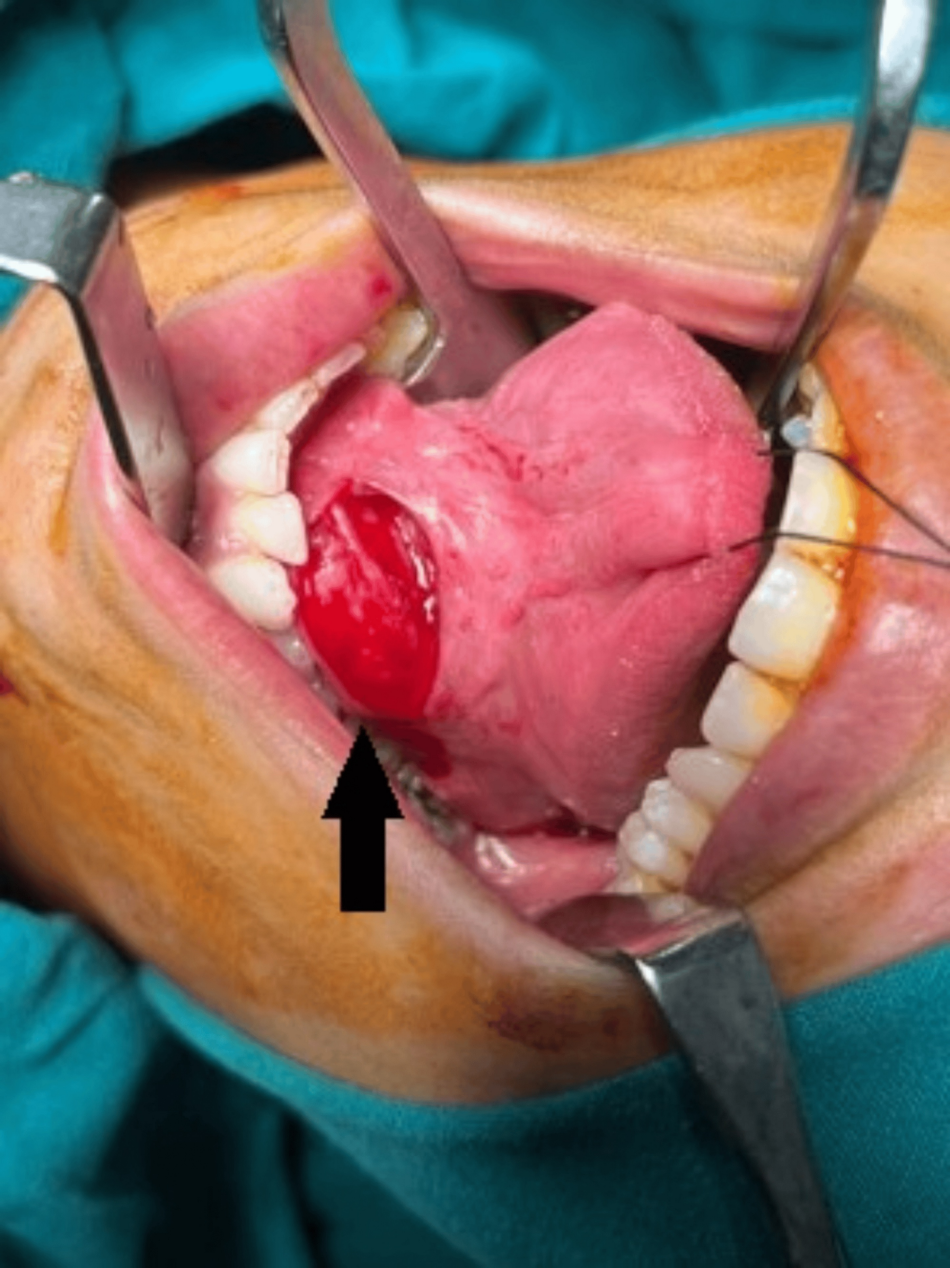
An intraoperative photo of the lesion An arrow indicating an incision over the lesion

**Figure 3:**
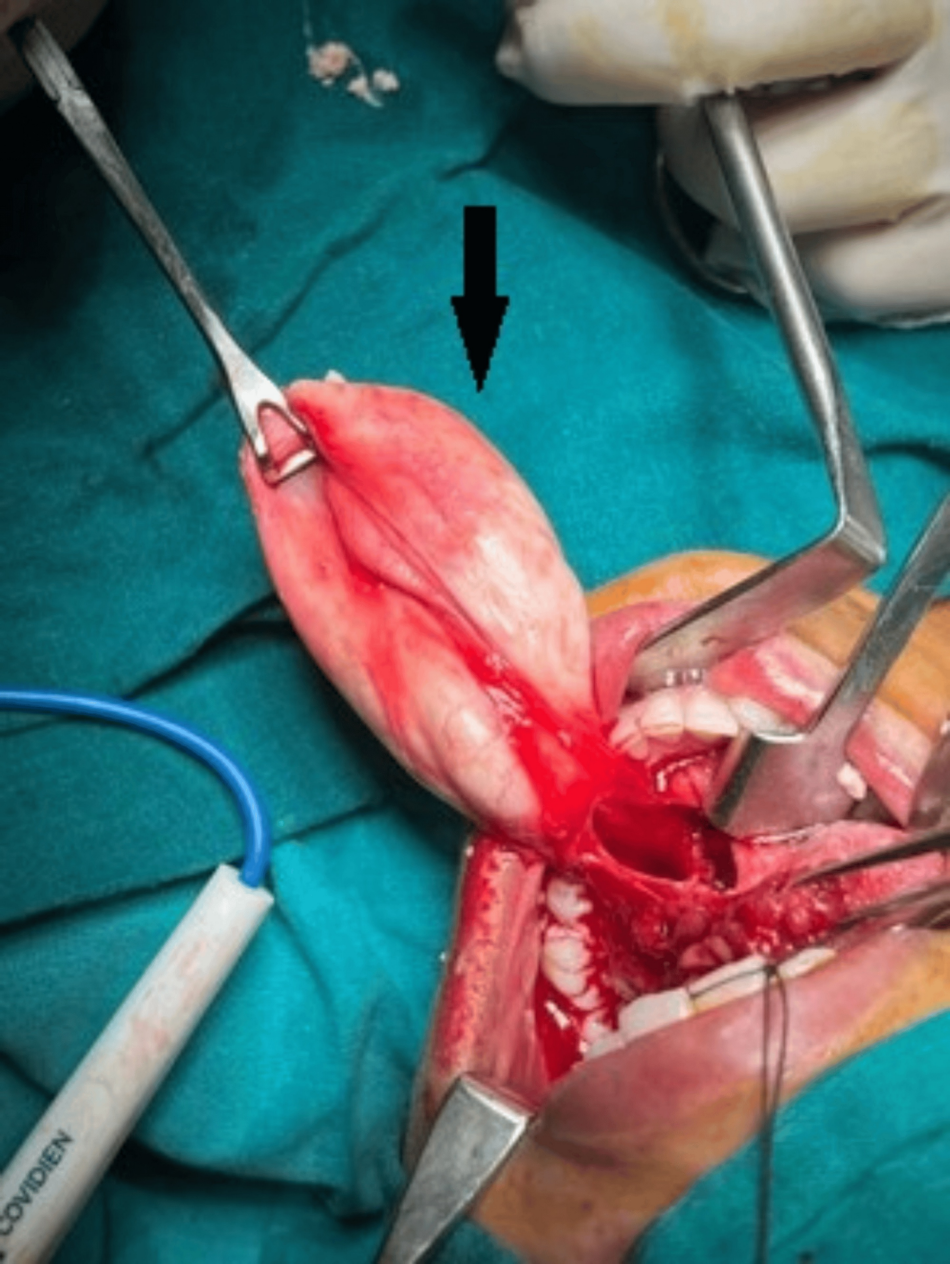
An intraoperative photo of the lesion The arrow shows the specimen removed in toto

**Figure 4:**
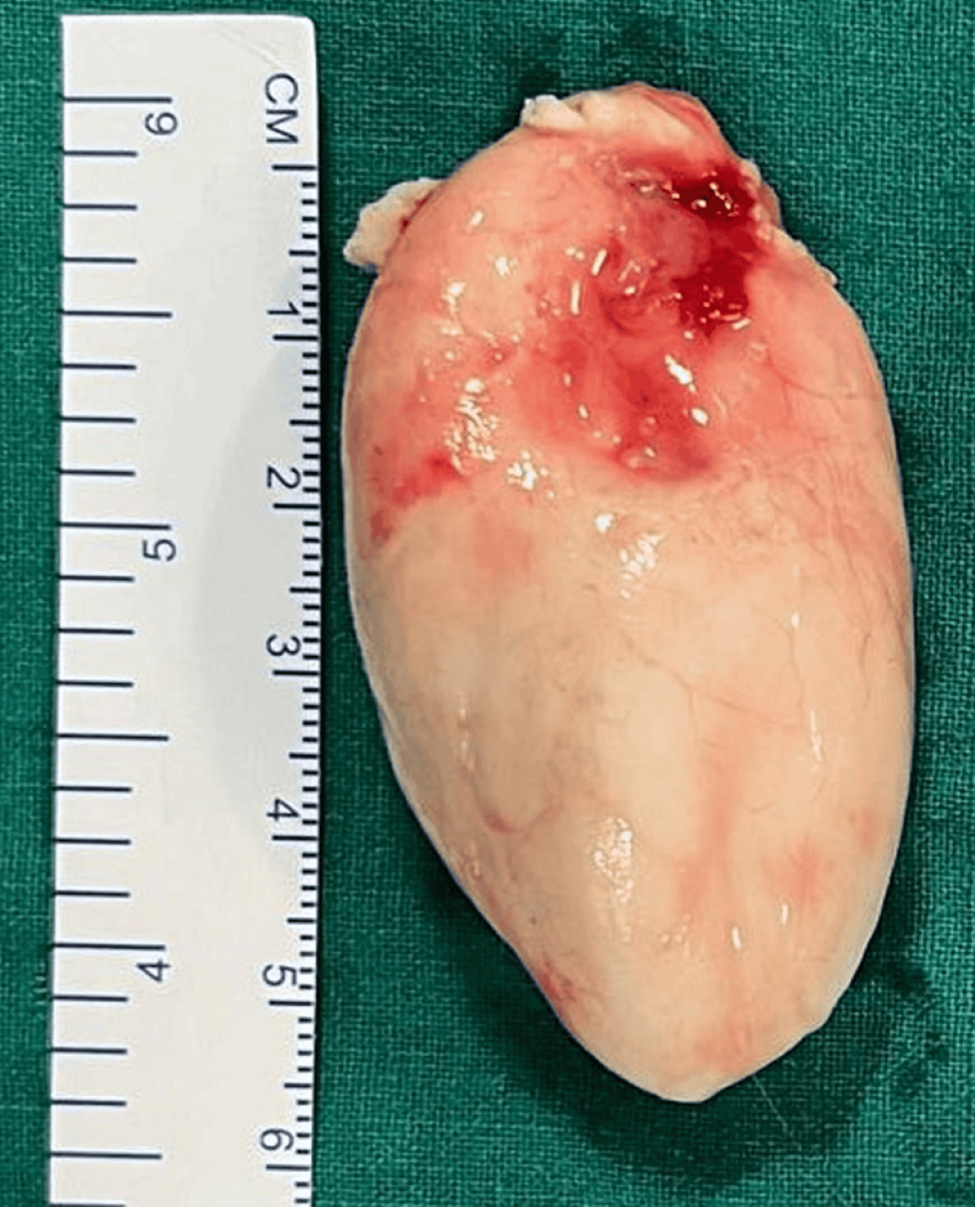
An intraoperative picture showing the excised specimen The picture shows an excised specimen of the cyst

A complete excision of the left sublingual dermoid cyst was performed. An incision was made on the swelling in the floor of the mouth (Figure 2), and the cyst was removed in toto (Figure 3). The histopathology report identified it as a dermoid cyst. Following surgery, the surgical wound (suture site at the floor of the mouth) was evaluated, and good oral hygiene was encouraged (Figure 4).

## Discussion

It is rather uncommon to see oral dermoid cysts in the head and neck region. The outside portion of the eyebrow is a common place in the head and neck to find dermoid cysts. These cysts usually start in the testes and ovaries [1].

In 1955, Meyer published the first description of the histological distinction between oral cysts. The cyst is called an epidermoid when it is only visible as epithelium in the lining; it is considered a dermoid when skin adnexa is found; and it is labeled a mixed cyst when it contains additional tissues, such as muscle, cartilage, or bone. People call it a teratoid. In the third and fourth weeks of embryonic development, during the midline fusion of the first and second branchial arches, epithelial cells become stuck and expand, resulting in developmental defects such as dermoid cysts on the floor of the mouth. The cutaneous appendages and epithelium that are traumatized or iatrogenically inserted give rise to acquired morphologies [5].

The look and functionality of the mouth floor may be impacted by benign growths called sublingual dermoid cysts, which grow gradually. It’s still unknown exactly why they form. There are multiple theories proposing that they originate from anomalies in development. These may include the failure of embryonic mesenchyme fusion [6]; the entrapment and growth of epithelial debris during the embryonic tongue’s midline fusion in the third and fourth weeks of intrauterine life [7]; traumatized epithelium implantation [8]; or the displacement of normally differentiated epithelial bands from their customary position [8].

Based on their histological features, Meyer divided the cysts in the floor of the mouth into three distinct entities: teratoid, dermoid, and epidermoid. However, all three types could be referred to as "dermoid" [9]. Histologically, a stratified squamous epithelium with a unique granular cell layer that is ortho-keratinized lines the inside of dermoid cysts. Keratin is often abundant in the lumen of the cyst. There are visible respiratory epithelial regions. Skin appendages that are a part of the fibrous connective tissue that makes up the cyst wall include hair follicles, sebaceous glands, and/or sweat glands.

Anatomically, dermoid cysts are categorized as either middle genio-glossal (supra-mylohyoid) or lateral, median geniohyoid (infra-mylohyoid) [5]. The present case involves a sizable median geniohyoid dermoid cyst. It is rare to see pure lateral dermoid cysts; these cysts usually occur in the sublingual, submental, and submandibular areas of the oral cavity [10]. Dermoid cysts can vary in size from 1.2 cm to 12 cm at their maximum dimension, per published studies [11]. Dimensions of 6 cm and above have been classified as huge. Size has an effect on the surgical technique, whether it is extraoral or intraoral [5].

The sudden expansion of the cyst is assumed to be related to the onset of puberty, which is linked to an increase in sebum production from the sebaceous glands [12].

Because there are variations in treatment, preoperative diagnosis is crucial. With careful investigation, the majority of cases can be clinically diagnosed. A traditional presentation has an indentation on light finger pressure, dough-like consistency, and midline symmetry. Simple-film imaging is not very useful for diagnosis. A cystic nature, size, and anatomical placement are demonstrated by CT, MRI, or USG, which are the main methods used to make the diagnosis [1,12,13]. MRI proved to be the most effective technique for accurately displaying location, extension, and demarcation in a study comparing several radiographic imaging techniques [14]. The diagnosis can also be made by Doppler-mode USG and fine needle aspiration cytology [10,15].

The tumor is surgically removed as part of the treatment. Small tumors are treated using the intraoral method; particularly large sublingual dermoid cysts impacting the submandibular and submental regions, as well as infections that may jeopardize the patient's airways, are treated with the extraoral approach [16]. This is due to concerns about inadequate access and the potential for recurrence. Second, there is evidence that salivary contamination and infections are risk factors for intraoral incisions. The review by Vélez-Cruz et al. found that there is a higher preference for intraoral techniques (59.3%) than extraoral approaches (30.5%). Just two of the 58 documented cases used a hybrid strategy [11].

General anesthesia is the recommended option since it allows for more patient comfort, retraction, and vision. Nasotracheal intubation is used to keep the tube out of the operative field. During the procedure, the patient is positioned supine. Large cysts can be removed with an intraoral technique that produces good functional and cosmetic effects, as this example shows. Wharton's ducts will be simpler to find if they are cannulated. When the cyst is clearly defined, an intraoral approach should be investigated, since it can offer access to sublingual and even submental areas without the need for a mandibular osteotomy or a skin incision in the submental fold.

Factors to consider encompass post-operative swelling, form, functionality, speech, taste, and the process of wound healing [17]. Recurrence is highly uncommon when the lesion is completely excised [18].

## Conclusions

Dermoid cysts located in the floor of the mouth are uncommon developmental abnormalities, constituting only 0.01% of all oral cysts. Because they are rare and present with non-specific symptoms, they are frequently misdiagnosed. An accurate differential diagnosis is crucial, as the recommended surgical approaches vary depending on the size and location of the cyst. Additionally, prompt treatment is essential due to the potential for breathing difficulties, swallowing issues, and recurrent respiratory infections in affected patients. Surgical removal is the preferred treatment, with the specific approach determined by the cyst's size and its relationship with the mylohyoid muscle. 
